# An Initial Approach to Increase Job Satisfaction Through Workplace Spirituality

**DOI:** 10.3389/fpsyg.2021.768290

**Published:** 2021-11-12

**Authors:** Ricardo Luiz Fernandes Bella, Osvaldo Luiz Gonçalves Quelhas, Fernando Toledo Ferraz, Douglas Vieira Barboza, Sergio Luiz Braga França

**Affiliations:** Department of Production Engineering, Fluminense Federal University, Niterói, Brazil

**Keywords:** workplace spirituality, job satisfaction, work psychology, spiritual factors, human factors

## Abstract

Job satisfaction is a widely discussed topic in work psychology, but what might be the contributions of recent discussions of workplace spirituality? This research allows a qualitative measure of workplace spirituality relevance by workforce perspective that can be reproduced in other organizations through a questionnaire application. The spiritual factors of the workplace were classified according to the Kano model that identifies the potential for actions and investments to be transformed into job satisfaction. In this application, it was identified that investments in the coherence and purpose of work factors can generate more than proportional satisfaction in the individuals of this organization. The identity, values, cohesion, meaning, and climate of work factors could generate a proportional satisfaction to the investments. The inner life and community factors cannot generate satisfaction, but when investment levels in these factors did not meet the expectations, it potentially generates dissatisfaction. Finally, investments in belonging, connection, and environmental factors were indifferent to the satisfaction level in this organization. The researchers also pointed out opportunities of investments to the organization.

## Introduction

Spirituality at work is a recent issue that has been growing over the last two decades. Its thematic axis develops mainly on the discipline of organizational behavior in the context of culture. Given the interdisciplinary content of behavior, contributions from different areas of knowledge are expected. Work psychology is one of the areas with the greatest potential to contribute to this topic of spirituality at work, as it combines knowledge about the perception of individuality and its relationships with the organizational environment.

The spirituality at work movement has been developing from growing demand from current generations. Within the agenda of movement, there is an expectation of maintaining their identity in all social roles that the individual plays (Thakur and Singh, 2016). Work is no longer seen as a career or a source of income and is understood as a means of personal development and the exercise of a greater purpose (Noor and Arif, 2011).

Currently, there are already spiritualized organizational cultures. Companies that articulate spirituality in the workplace understand that employees have an inner life that is nurtured by meaningful work done within the context of a community (Duchon and Plowman, 2005). In these companies, there is freedom for people to express their tastes, feelings, and individuality (Kumar and Kumar, 2014).

Companies ultimately note that their ability to add and retain talent is conditioned on their ability to meet the needs of the target workforce (Ramnarain and Parumasur, 2015). Thus, leading companies in their markets have been intensifying actions and investments to build a work environment that promotes satisfaction and quality of life (Osman-Gani et al., 2013). For an organization to present efficient and effective results, it must meet the demands of the organization and stakeholders in a systemic and integrated manner (Barboza et al., 2020). Workplace spirituality is one of the growing strategies in organizational designs to remain attractive, competitive, and sustainable (Al-Hosaini and Sofian, 2015).

## Background

### Work Psychology and Job Satisfaction

Organizational and work psychology merges the study of two fields to reconcile the understanding of the relationship between individuals and organizations. From the perspective of psychology, themes such as motivation, cognition, learning, and emotions are studied to understand the bonds of the individual with his/her work and the organization in which he/she works (Ostroff et al., 2003).

Among the main links addressed by work psychology, there were satisfaction, involvement, and organizational commitment. Bringing basic definitions: satisfaction is the general feeling of a person about their work, being an indicator of occupational health; involvement is the degree of identification of the individual with the work, being an indicator of personal valuation; commitment is the degree of identification with the organization and its objectives, being an indicator of job stability (Whitman et al., 2010).

Within the organizational perspective, work psychology studies themes such as power, leadership, culture, and socialization to understand the structures that permeate work. Based on the combined knowledge of psychology and organizations, this area of knowledge seeks to explain, predict, and model organizational behavior, focusing on four key dependent variables for organizational performance: productivity, turnover, absenteeism, and job satisfaction (Wegge et al., 2007).

Job satisfaction has received a lot of attention from executives due to the popularity of the premise that satisfied workers are more productive. However, despite companies with higher levels of satisfaction being the most productive on average, satisfaction does not generate productivity, but work does generate satisfaction (Locke, 1976). So, that is important for organizations to offer jobs that are both stimulating and intrinsically rewarding. And workplace spirituality could be a path to design these jobs.

### Workplace Spirituality

The spiritual approach can be interpreted as more than a subjective perception of life, but from the experience of working through three structures that are self, others, and collectively oriented (Bella et al., 2018). The harmonization of these three dimensions results in what Ashmos and Duchon (2000) define as work spirituality: “the recognition of an inner life that nourishes and is nourished by meaningful work which takes place in the context of community.”

For this, it is necessary to make practices that fulfill the spiritual needs to intentionally achieve a spiritual work environment (Doram et al., 2017). The main strategies that current enterprises are using to deal with spirituality are through leadership and organizational culture (Fite et al., 2011). Nevertheless, organizational efforts are still unplanned even with increasing evidence of their contributions (Houghton et al., 2016).

Some case studies on spiritual human factors take place in hospitals because of their circumstances that lead to reflection on existential issues (Sani et al., 2016). Other studies, more recent, are taking place in organizations with a culture of result orientation due to their strong leadership and team spirit environment (Chawla, 2016).

A considerable number of reviews describe multiple aspects of spiritual factors, but to increase job satisfaction, it is required a structured approach for these aspects. Bella et al. (2018) organized 12 spiritual human factors through a systematic literature review to fill out this gap. In Table 1, these 12 factors are shown.

**TABLE 1:** Workplace spiritual factors.

**Dimension**	**Attribute**	**Description**
Inner life and identity	Identity	Experience an integral self-awareness (i.e., physical, emotional, spiritual, and social)
	Belonging	Experience belonging and connection with you, others, and the universe
	Values	Experience the employment, by you and your colleagues, of your values at work
	Inner life	Experience zeal for internal issues (e.g., feelings, beliefs, and values)
Purpose and meaning	Coherence	Community: the necessity for reciprocity and respect for individuality
	Cohesion	Experience challenges with gradual evolution
	Purpose	Experience challenges with gradual evolution
	Meaning	Experience alignment of your vocation and sources of job satisfaction (i.e., apply talents and qualities at work)
Community and connection	Climate	Experience a mild organizational climate (i.e., promoting autonomy, communication, and cooperation)
	Community	Experience respect for basic beliefs and values
	Connection	Experience personality in work relationships (i.e., high-quality relationships)
	Environment	Experience an organizational environment open to the expression of personality in an integral way

*Source: Bella et al. (2018).*

Therefore, the long-term psychological consequences of coronavirus disease 2019 (COVID-19) should be considered (Bella et al., 2021). The workplace may represent an opportunity to integrate occupational health practices with public health activities (Chirico and Ferrari, 2021). During the current pandemic, higher levels of spiritual distress have been associated with lower mental health levels in the population (Jakovljevic et al., 2019). Spiritual beliefs during the COVID-19 pandemic have also been associated with higher levels of hopefulness which suggests that spirituality can be used by individuals in a positive way (Van Der Walt and De Klerk, 2014; Chirico, 2021).

Despite the methodological difficulties of spirituality researches (Weathers et al., 2016), there is agreement that spirituality is not limited to religiosity (Tejeda, 2015; Chirico et al., 2020) and could be applied in organizations (Steinhorn et al., 2017). However, Chirico et al. (2019) have shown that workplace violence was prohibited just if it involves moral or religious offense in many countries.

## Materials and Methods

### Research Approach

This research builds a qualitative measurement instrument through a quantitative approach. Qualitative measurement takes place through the categorization of the studied human factors, that is, after the analysis, categories are assigned to the studied factors. The categories used in the study are predetermined by the chosen model, the Kano model. Analyzes, in turn, use a quantitative approach, as they calculate the frequencies of observed data through coefficients established by the same model.

Data are qualitative, representing the perception of individuals participating in the research. The perceptions captured concern the human factors of spirituality at work previously established by Bella et al. (2021). These factors represent the qualitative variables verified by a closed questionnaire with questions and answers planned according to the Kano model. The measurement scale used derives from this same model.

The population studied comes from an intentional sampling from the collaboration of a company in the naval industry. In all, approximately 100 questionnaires representing all employees of this organization were sent. The response rate was 70% from which all data were processed to propose the results found.

### Research Instrument

Kano’s model (Kano, 1984) was used to create a questionnaire. Based on this model, a couple of questions were presented for each factor. The first question is concerned with participant reaction when the factor is present (functional form). The second question is concerned with participant reaction when the factor is absent (dysfunctional form).

The functional forms were presented by the sentence “How do you feel when you find a sense of (variable) at work?” The dysfunctional forms were negative of these sentences. These questions were answered in a five-point scale provided by Kano’s model, which are like, must-be, neutral, live-with, and dislike. So, for each question, the respondents must choose one of these five options.

Kano’s model is traditionally used to assess the quality of products and services, but it could be used to address workplace characteristics and map job satisfaction about spiritual factors due to a theoretical parallel created between two systems: the usually analyzed system, consumer-attribute-product; and the system of this research, worker model of spirituality workplace. In the original context, the model intends to explain the behavior trend of consumers to the characteristics of a product through the relationship between two variables: consumer satisfaction and the functionality of a product attribute. In this research, worker satisfaction is related to the attributes of the work environment.

### Sample and Data Collection

The results consider a sample of 70 people from an offshore company with approximately 100 employees. To achieve this sample, the support of the board of directors was fundamental. The period of questionnaire application lasted for 2 weeks, but most answers were collected in the first week. It should be remarked that the invites were sent through the intranet by human resources support; however, the entire registry was maintained by the researchers for ensuring anonymity.

In addition, some points can be highlighted from this sample, namely, the average age between 26 to 30 years; the prevalence of male gender representing 70% of the sample; the time of professional activity with the highest concentration in the range of fewer than 5 years; and prevalence at the operational level. In Table 2, the sample characteristics are shown.

**TABLE 2:** Sample characteristics.

**Biographical characteristics**	**Sample sets distributions**				
Age (years)	16–20	21–25	26–30	31–35	Over 35
Number of individuals	4 (6%)	14 (20%)	28 (40%)	13 (18%)	11 (16%)
Gender	Male	Female			
Number of individuals	49 (70%)	21 (30%)			
Professional activity time (years)	Under 5	06-10	11-15	Over 15	
Number of individuals	30 (43%)	20 (28%)	15 (21%)	5 (8%)	
Hierarchical level	Operational	Management	Strategic		
Number of individuals	46 (66%)	17 (24%)	7 (10%)		

To check the responses to the questionnaire, see Supplementary Appendix.

### Data Analysis

Kano’s model was used for data analysis. This model analyses the relationship between two variables: functionality and satisfaction. Combining these two variables, the model can explain the tendency of satisfaction based on the functional performance of a factor. The tendencies of satisfaction behavior were previously mapped by some categories as:

• Must-be (M): A factor classified in this category consists of the basic criteria, and it will be extremely dissatisfied if it is not fulfilled. However, its fulfillment does not increase satisfaction level because it was taken for granted.
• One-dimensional (O): The presence of a factor in this category will increase the satisfaction level, while its absence will proportionally decrease the satisfaction level.
• Attractive (A): A factor falling in this category generates absolutely positive satisfaction, while customers will not be dissatisfied at all when it is not fulfilled.
• Reverse (R): A factor falling in this category should be removed because its functional presence is harmful to satisfaction, but its dysfunctional absence is beneficial.
• Neutral (N): A factor in this category does not contribute much to satisfaction irrespective of whether it is fulfilled or unfulfilled.
• Questionable (Q): This outcome indicates that the question was described incorrectly, or an illogical response was given by the respondent.

To get into these categories, it is necessary to combine the responses of functional and dysfunctional questions about the analyzed factor. In Table 3, the factors classification for an individual is presented by different answers combinations.

**TABLE 3:** Spiritual human factors classification for an individual.

**Functional answer**	**Dysfunctional answer**				
	**Like**	**Must-be**	**Neutral**	**Live-with**	**Dislike**
Like	Questionable	Attractive	Attractive	Attractive	One-dimensional
Must-be	Reverse	Indifferent	Indifferent	Indifferent	Must-be
Neutral	Reverse	Indifferent	Indifferent	Indifferent	Must-be
Live-with	Reverse	Indifferent	Indifferent	Indifferent	Must-be
Dislike	Reverse	Reverse	Reverse	Reverse	Questionable

To consolidate the answers of various respondents, two coefficients provided by Berger (1993) were used—the satisfaction coefficient (SC) and the dissatisfaction coefficient (DC). These coefficients indicate the percentage of respondents who were satisfied with the presence of a factor and the percentage of respondents who are dissatisfied with the absence. They were calculated, Equations 1, 2, considering the abbreviations of previous categories:


(1) $$SC=\frac{\%A+\%O}{\%A+\%O+\%M+\%N}$$

(2) $$DC=\frac{\%O+\%M}{\%A+\%O+\%M+\%N}$$

**FIGURE 1:**
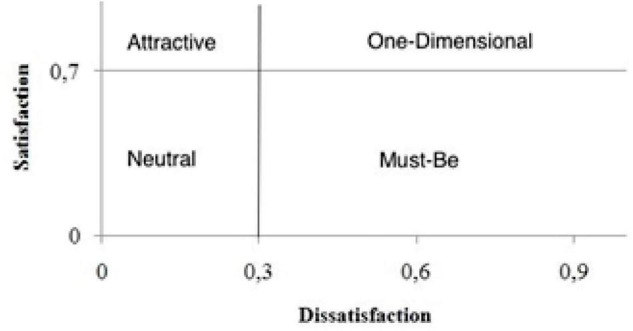
Spiritual human factors classification by coefficients.

For the interpretation of the coefficients, it is interesting to use a graphical analysis. Two parameters were used to make four quadrants that represent the categories of the model. These parameters separate the quadrants according to better results interpretation. In this case, the percentage of 30% of unsatisfied respondents was considered to separate quadrants and 70% for satisfied respondents. Thus, the classification of factors can be arranged according to the following quadrants shown in Figure 1.

According to the percentage character of the coefficients, the result varies within a range between 0 and 1 for satisfaction and between 0 and −1 for dissatisfaction. Result analysis can be performed using graphical or interval analysis. Graphical analysis occurs by positioning the element in one of the quadrants formed by dividing the intervals. The division of intervals depends on the rigor of the analyst for classifying the elements. So, this research used a more rigorous range of 0.7 for the satisfaction coefficient and −0.3 for the dissatisfaction coefficient.

## Results

### Overview

**FIGURE 2:**
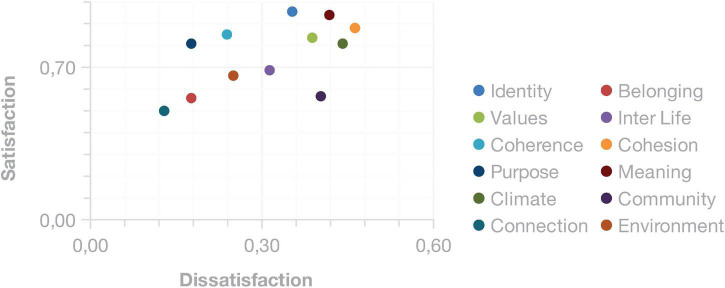
Factors classification for the organization.

The results overview brings the consolidated classification for all spiritual factors analyzed. In Figure 2, the map of workplace spirituality of the studied sample can be observed. In this figure, we have four quadrants indicating the four interest categories: attractive, one-dimensional, must-be, and neutral. The 12 human factors were classified into these four categories giving the scenario of the company spirituality.

The coherence and purpose of factors were classified as attractive, so practices that meet these expectations should generate more than proportional satisfaction. The identity, values, cohesion, meaning, and climate of factors were classified as one-dimensional, so the attention given to them generates proportional satisfaction. The inner life and community factors were classified as must-be, so for these elements, there is a requirement, which when not met generates dissatisfaction. Finally, the belonging, connection, and environmental factors were classified as neutral, so they are indifferent to the satisfaction level. To check the results, see Supplementary Appendix.

### Attractive Factors

The spiritual factors classified as attractive generate more than proportional satisfaction when they are attended. On the other hand, these are considered differential, because when they are not met, they do not generate dissatisfaction. On these spiritual needs, there is no expectation of care; however, a potential value is perceived in them. Thus, it is strategically preferable to invest first in must-be and one-dimensional factors before paying attention to attractive factors. In Table 4, the spiritual factors classified as attractive for different hierarchical levels are presented.

**TABLE 4:** Attractive factors for the organization and its levels.

**Organization**	**Operational level**	**Management level**	**Strategic level**
Coherence	Purpose	Coherence	Coherence
Purpose			Purpose
			Values

The coherence factor refers to the level of difficulty of the tasks, whether they are suited to personal skills and with a defined scope. This need manifests itself at an institutional level, in the relation between individual and organization, and can be approached through attention by the leadership when delegating tasks.

The purpose factor concerns the sense of daily commitment to a legacy that integrates life. This need manifests itself in the interpersonal scope, in the relation of collaborator to collaborator, being able to be approached through the attention to metrics that highlight the own contribution to the team.

### One-Dimensional Factors

The spiritual factors classified as one-dimensional generate satisfaction proportional to the attention given to this spiritual need. This classification refers to a cost-benefit-oriented expectation, so the lack of attention to these factors also generates a proportional dissatisfaction with the dysfunction. So strategically once you have met the must-be factors, it is interesting to invest in the one-dimensional. For a marginal issue (economic concept), the cost tends to continue growing, while the benefit decreases. Thus, before it reaches the point of marginal effect, it is interesting to invest in the attractive factors.

In Table 5, the spiritual factors classified as one-dimensional are presented according to the analysis in different hierarchical levels. In this table, we can see that a great number of factors were identified as one-dimensional, mainly by the strategic level that marked nine of the 12 factors. However, in the general context, only five were classified: identity, cohesion, meaning, climate, and values.

**TABLE 5:** One-dimensional factors for the organization and its levels.

**Organization**	**Operational level**	**Management level**	**Strategic level**
Identity	Identity	Identity	Identity
Cohesion	Cohesion	Cohesion	Cohesion
Meaning	Meaning	Meaning	Meaning
Climate	Climate	Climate	Climate
Values	Values	Values	
	Inner life		
	Coherence		
	Community		
	Environment		

The identity factor concerns the development of integral self-knowledge. This need manifests itself at the intrapersonal level, in the relation of the individual to the individual, and can be supported by the organization through courses, mainly emotional intelligence that allows identifying the natural mechanisms of functioning of the mind itself.

The cohesion factor addresses challenges in all spheres by considering the gradual evolution and alignment with purpose. This need develops intrapersonal and can be supported by the promotion of dialog in pairs or groups.

The meaning factor refers to the recognition of the alignment of their vocation (the most outstanding talents and qualities) and the sources of job satisfaction. This need manifests itself at the interpersonal level and can be addressed through behavioral analysis to identify individual strengths.

The climate factor refers to the lightness in the organizational climate. This need manifests itself intrapersonal and can be addressed through management that empowers employees to dialog, proactivity, and cooperation.

The values factor refers to the recognition of the use of their personal values at work. This factor manifests itself interpersonally and can be approached through autonomy to define values at the team level.

### Must-Be Factors

The spiritual must-be factors are considered as basic needs, so when they are not attended, they generate dissatisfaction; however, when they are attended, they are not able to generate satisfaction. In this way, the must-be spiritual factors are the minimum expected for the work environment and strategically should not be neglected. Among all the factors classified so far, the must-be has priority over the others given the negative repercussions of not attending them.

An interesting question about the must-be factors is that this was the group with the lowest number of factors classified. This result indicates that spiritual factors are not generally understood as basic needs. This fact corroborates the thinking of the hierarchy of Maslow needs, where the higher-order needs are more difficult to be perceived as such, due to a latency of the lower-order needs. However, the few factors identified as must-be should not be neglected, given the potential for dissatisfaction generated by their non-compliance. In Table 6, the must-be spiritual factors are presented.

**TABLE 6:** One-dimensional factors for the organization and its levels.

**Organization**	**Operational level**	**Management level**	**Strategic level**
Community	Connection	Community	Community
Inner life		Inner life	

The community factor talks about respect for the basic beliefs and values of everyone. This need manifests itself at the institutional level and can be addressed through the formation of leadership.

The inner life factor is about zeal for internal issues. This factor is manifested in the intrapersonal scope and can be approached through training with positive psychology, mainly through the cultivation of positive perspectives.

### Neutral Factors

Spiritual factors regarded as neutral do not contribute to employee satisfaction or dissatisfaction. Regardless of the level of attention given to these needs, the employees will hardly perceive it as any contribution to them, being disregarded as relevant factors for them. Strategically, it is not interesting to invest in factors classified as neutral. In Table 7, the neutral spiritual factors are presented.

**TABLE 7:** Neutral factors for the organization and its levels.

**Organization**	**Operational level**	**Management level**	**Strategic level**
Belonging	Belonging	Belonging	Belonging
Connection	Connection	Connection	Connection
Environment	Environment	Environment	Environment
		Purpose	Inner life

The belonging factor concerns the perception of a connection with yourself, with other people, and with the universe. This need manifests itself in the institutional locus and can be addressed through engagement in themes such as sustainability and spirituality. This result is noteworthy because it is the only factor that presented some indication of religion and was classified at all levels as indifferent, indicating a probable expectation of separation between religion and work.

The connection factor refers to the personality in labor relations. This need manifests itself at the interpersonal locus and can be addressed through team engaging practices, such as experiential training and fellowship.

The environment factor refers to the openness of the organizational environment to the expression of personality. This need is manifested at the institutional level and can be addressed through the relaxation of space in terms of norms in general or even in the characterization of the environment in more welcoming places.

## Conclusion

This article takes a pragmatic approach to workplace spirituality to propose a questionnaire that maps job satisfaction tendencies based on spiritual human factors. The starting point was the systematic literature review of Bella et al. (2018) about workplace spirituality that identifies the main spiritual factors. After that, the questionnaire was designed based on Kano’s model to achieve an orientation for better conditions to work.

The results showed that human spiritual factors can be differentiated among the categories predefined by Kano’s model. The three dimensions of spirituality were analyzed resulting in different priorities for the different hierarchical levels of the organization. Most factors were classified in the one-dimensional category that generates a satisfaction proportional to the attention given to them. On the other hand, the must-be category was the one with the lowest number of factors classified, identifying that the spiritual needs are perceived as a differential to the workforce.

Finally, this model can also contribute to individuals in the matter of understanding that their spiritual health needs attention as well as their body and mind. Besides that, the main application of this model is for workplace design. For example, the spiritual human factors can be used in strategic planning at human resource practices.

## Data Availability Statement

The original contributions presented in the study are included in the article/Supplementary Material, further inquiries can be directed to the corresponding author/s.

## Ethics Statement

Ethical review and approval was not required for the study on human participants in accordance with the local legislation and institutional requirements. The patients/participants provided their written informed consent to participate in this study.

## Author Contributions

OQ: elaboration of the initial concept and introduction. FF: literature review and method review. RB: methodological development and results. DB and SF: conclusion and final review. All authors contributed to the article and approved the submitted version.

## Conflict of Interest

The authors declare that the research was conducted in the absence of any commercial or financial relationships that could be construed as a potential conflict of interest.

## Publisher’s Note

All claims expressed in this article are solely those of the authors and do not necessarily represent those of their affiliated organizations, or those of the publisher, the editors and the reviewers. Any product that may be evaluated in this article, or claim that may be made by its manufacturer, is not guaranteed or endorsed by the publisher.
