# Supplementary material for: An Initial Approach to Increase Job Satisfaction Through Workplace Spirituality
Source: Front Psychol. 2021 Nov 12;12:768290. doi: 10.3389/fpsyg.2021.768290 (PMC8632702; doi:10.3389/fpsyg.2021.768290)
Supplement: Supplementary file 1 [file Data_Sheet_1.pdf]

SUPPLEMENTARY MATERIALS

1. Consent Form (Ethics and Information Security)

A consent form for the research was signed between the parties, that is, the researcher and organization. In this term, ethical conduct was guaranteed in relation to the information collected, mainly in relation to information security. Among the points clarified in the consent and participation term, the following stand out:

- Study objective – to address the issue of non-disabling mental health disorders, recognized as anxiety, stress and depression, through attention to spirituality in the workplace in order to propose a model for a strategic approach to spirituality in organizations;
- Procedures – the company's participation in this survey will consist of conducting the questionnaire set up for the survey via intranet by a manager from the human resources area. An interview will also be held to assess and analyze the results with the people manager. The questions in the questionnaire applied to employees were related only to the theme of spirituality at work and demographic variables to identify the profile of the sample. The interview will serve to present and validate the data, as well as verify the manager's perception of the potential contribution of the research findings;
- Benefits – this research will bring greater knowledge about the topic addressed, without direct benefit to the company, not committing the researcher to any obligation arising from data collection. However, the researcher is committed to making an executive presentation of the collected data and their analysis, as well as providing a digital copy of this thesis;
- Risks – filling out the questionnaire does not pose any physical or psychological risk to employees. The right of non-participation of its collaborators will be guaranteed, as well as the clarification regarding any doubts about the research instrument;
- Confidentiality – at no time will the identity of the people who participated in the survey be disclosed, not even to the company's managers, ensuring total anonymity of the survey participants.

2. Questionnaire Structure

Four sections organize the elements of the questionnaire.

Section 1: Sample Profile (Socio-Metric Data)

- Age: 16 to 70 years, grouped every 5 years;
- Gender: Male or Female;
- Schooling: from Fundamental to PhD;
- Professional Activity Time: from no up to 35 years, grouped every 5 years.

Section 2: Inner Life and Spiritual Identity

This section begins with the following presentation: This dimension of Spirituality at Work concerns the need for some people to cultivate an Inner Life, or the Inner Self, which is often understood to be their Soul or Spiritual Identity. After this presentation, the following questions are presented:

- How do you feel when there is opportunity to develop self-knowledge?
- How do you feel when there is opportunity to develop a connection with your god?
- How do you feel when there is opportunity to employ your personal values at work?
- How do you feel when there is an opportunity to care for internal personal issues?

Section 3: Meaning and Purpose at Work

This section begins with the following presentation: This dimension of Spirituality at Work concerns the need for some people to recognize or understand a Significance or Major Purpose at work, which often needs to reflect or be connected with Personal Values. After this presentation, the following questions are presented:

- How do you feel when the tasks fit your personal skills?
- How do you feel when you face professional challenges with a gradual evolution?
- How do you feel when you have the opportunity to commit to a legacy that integrates all areas of your life?
- How do you feel when there is opportunity to develop your strengths at work?

Section 4: Sense of Connection and Community

This section begins with the following presentation: This dimension of Spirituality at Work concerns the need of some people to feel belonging or Connected to a Community, which is often reflected in the need to be accepted through the expression of their Inner Life. After this presentation, the following questions are presented:

- How are you feeling when there is a light organizational climate?
- How do you feel when there is concern about each individual's core beliefs and values?
- How do you feel when you are in work relationships?
- How do you feel an open organizational environment in the expression of authenticity?
